# Development of a Wireless Sensor Network for Individual Monitoring of Panels in a Photovoltaic Plant

**DOI:** 10.3390/s140202379

**Published:** 2014-01-30

**Authors:** Miguel J. Prieto, Alberto M. Pernía, Fernando Nuño, Juan Díaz, Pedro J. Villegas

**Affiliations:** Área de Tecnología Electrónica, Universidad de Oviedo, Edificio Departamental No. 3. Campus Universitario, 33203 Gijón, Spain; E-Mails: amartinp@uniovi.es (A.M.P.); fnuno@uniovi.es (F.N.); jdiazg@uniovi.es (J.D.); pedroj@uniovi.es (P.J.V.)

**Keywords:** photovoltaic power systems, energy storage, supercapacitors, wireless sensor networks

## Abstract

With photovoltaic (PV) systems proliferating in the last few years due to the high prices of fossil fuels and pollution issues, among others, it is extremely important to monitor the efficiency of these plants and optimize the energy production process. This will also result in improvements related to the maintenance and security of the installation. In order to do so, the main parameters in the plant must be continuously monitored so that the appropriate actions can be carried out. This monitoring should not only be carried out at a global level, but also at panel-level, so that a better understanding of what is actually happening in the PV plant can be obtained. This paper presents a system based on a wireless sensor network (WSN) that includes all the components required for such monitoring as well as a power supply obtaining the energy required by the sensors from the photovoltaic panels. The system proposed succeeds in identifying all the nodes in the network and provides real-time monitoring while tracking efficiency, features, failures and weaknesses from a single cell up to the whole infrastructure. Thus, the decision-making process is simplified, which contributes to reducing failures, wastes and, consequently, costs.

## 1. Introduction

The high volatility of oil prices, coupled with increasing worldwide concerns over CO_2_ emissions, has led to the evolution of renewable energy concepts over the past few years. Among others the use of solar photovoltaic (PV), has emerged as the most appropriate solution and has continuously been gaining considerable attention among industry players all around the globe. With the growing demand for clean energy sources, the manufacture and deployment of solar PV cells and photovoltaic arrays have expanded dramatically in the recent years.

Monitoring of photovoltaic plants and optimization of the energy they produce is a key issue in order to guarantee this type of plants can serve to their goal of significantly contributing to supply the ever increasing demand of electrical power. Monitoring systems currently available on the market are limited to the evaluation of the average power produced in a given time interval, as a function of several parameters coming from the inverter [1–6]. Even though relevant, knowing the average power is not enough to either globally evaluate the overall performance of the PV plant or to identify potential failures affecting some plant components (such as single cells or clusters of cells). Instead, it would be very interesting to have information related to how much energy each of the PV panels in the plant is producing. Thus, it would be possible to easily identify the elements that are not operating properly and repair or replace them. Obviously, this would require that each of these PV panels be individually monitored and that the data obtained be communicated to some kind of control system, and here is where wireless sensor networks come into play.

A wireless sensor network (WSN) consists of several small, inexpensive sensor nodes that communicate detected events wirelessly [7–9]. Quite often, WSNs are used to monitor certain delicate situations as those indicated in [10]: floods [11], volcanoes [12], radioactive materials [13], wildfire [14], ethylene [15], methane [16], *etc.* However, they can also be used in other less critical cases, like the one covered in this work, where the information to be gathered is so exhaustive that several simple sensors (from a few tens to some hundreds) are needed. In these cases, it is the information flow that becomes critical, and several studies have been carried out covering this topic [17–19]. Smart sensor nodes with at least two sensors will be attached to each PV panel in order to monitor its performance. As well as the sensors, these nodes include a processor, a radio system and a power supply that provides the energy required by the node. This latter element is an important component in the correct operation of any smart wireless node.

Through the development of a WSN, the work presented in this paper supports deeper monitoring of relevant parameters to identify efficiency features, failures and weaknesses of the whole PV infrastructure. Data coming from the sensor network will be elaborated in order to simplify the decision making process and to help reducing failures, waste and, consequently, costs.

The paper is organized as follows: Section 2 summarizes the current state of the art and presents an overview of the system proposed in the paper. Section 3 describes the implementation of the system, paying special attention to the power supply developed and the communication protocol. Section 4 describes how the overall system operates and explains the algorithm of network routine. Experimental results are presented in Section 5 and final conclusions are provided in Section 6.

## 2. Overview of the Proposed System

The high costs of electricity produced from the sun highlight the importance of optimizing system operation, energy production and reliability. As a result, it is essential to analyze data and outputs of a photovoltaic (PV) plant in order to enable the elaboration of detailed and precise evaluation of the system performance and, in particular, of the effective energy production with respect to the plant's potential.

As previously stated, monitoring systems currently available on the market are generally based on data coming out from the inverter, which is a fundamental component of the plant and allows direct current to be transformed into alternating current and, therefore, into current entering the electrical grid. Generally, PV plants have an inverter every ten or fifty solar panels. The main parameters available are usually: instant power, frequency, voltage, electric current, environment temperature, inverter temperature, *etc.*

Each plant has a nominal power value depending on its characteristics (dimension, technologies, *etc.*), but also on the climatic conditions, particularly sun irradiation. Measurements from the inverter allow us to calculate the average power produced in a given time interval: day, week, year, *etc.*; However, although relevant, average power is not sufficient to detect whether the plant production is the effective optimum one or if some plant components (one or more solar cells) are affected by failures. In fact, average power significantly depends on some other parameters such as atmospheric pressure, wind speed and sun incident radiation. As an example, a full sunny day with dirty panels or a cloudy day with perfect clean panels could produce the same quantity of energy.

In the last years, significant developments have taken place in some technologies which can be usefully applied to the management of a PV plant, namely:

- Sensors and wireless sensor networks.
- Micro-inverters and electronic on-board of each panel.

These technologies allow users to implement new and powerful monitoring systems capable of optimizing the efficiency of each panel as well as that of the whole plant, while detecting faults or critical events. Several works can be found in literature related to remote monitoring of solar power plants [20–23], but not many go as far as trying to determine the performance of single panels [24].

The conceived system aims to overcome the limitations indicated above by designing a WSN to monitor the efficiency of a photovoltaic plant and optimize the energy production. Through this network real-time monitoring of some relevant parameters, such as electrical characteristics, critical parameters of the plant, sun irradiation or meteorological and ambient parameters for each panel, is carried out. Monitoring and analysis of these parameters will make the system suitable to carry out relevant services as described:

- Complete reports about production cycles supporting a set of tools to plan the activity (*i.e.*, maintenance) and the energy production.
- Surveillance system based on sensors, thus improving the security of the plant.
- Automatic signaling alerts to let operators know about failures or intrusions through different communication strategies: audio alerts, SMS, *etc.*
- Real-time monitoring of the effective energy power produced by each solar panel and, obviously, by the entire plant, according to the sun radiation and meteorological conditions.
- Measuring solar radiation in real time to optimize the exposition of panels with respect to sun irradiation.
- Estimating, through historical data stored by the system and weather forecast day per day, the production of energy.
- Specific and real-time maintenance through the individualization of eventual breakdown in short time.

In short, a solar power plant equipped with the system described in this paper can optimize energy production based on the surrounding ambient state. Nowadays, despite the relevant refinement of production technologies during the last years, many plants still don't have any optimizatizon of their energy production, resulting in an overall efficiency that is lower than the real capability of the plant.

**Figure 1.**
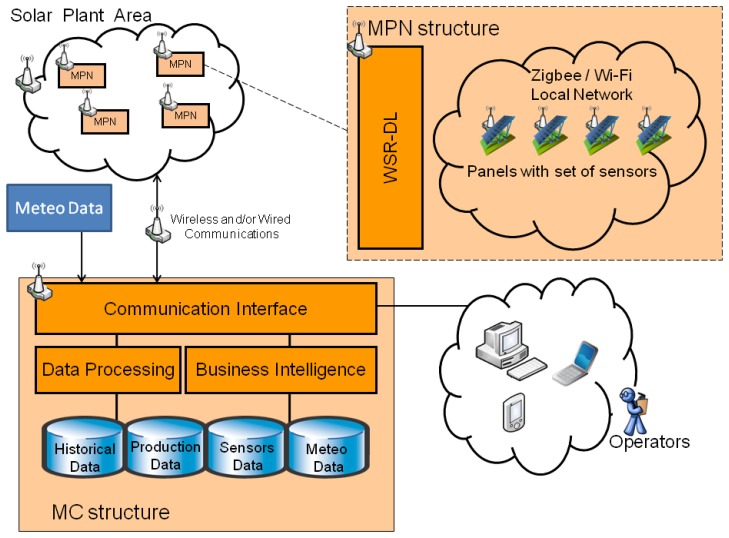
General block-diagram of the system framework.

To achieve such an ambitious goal, a system infrastructure has been developed made up of the following modules (Figure 1):

(1). A *Monitoring Center* (MC Structure in Figure 1) receiving real time data and images from sensors in the photovoltaic cells and from other sources (meteorological and solar radiation). This *Monitoring Center* integrates a set of tools to monitor production as well as to detect failures and to send alerts through different communication channels (PC, phone, smartphone, *etc.*). Additionally, it can estimate, through an innovative integration of data analysis (automatically stored by the system) and weather forecast, the potential production of energy power The *Monitoring Center* is also equipped with a surveillance system against thefts and actions of vandalism, and might also be able to control the orientation of the solar cells in order to maximize the energy production.
(2). A wireless network integrating wireless sensors by means of a Zigbee-based protocol (*Monitoring Plant Network*, MPN structure in Figure 1). This network is in charge of collecting relevant data and sending them to the *Monitoring Center*.
(3). A *Monitoring Environment* composed by sensors measuring physical parameters of the plant and of the single solar cell, but also environmental parameters such as, for example, effective solar radiation.

The *Monitoring Plant Network* has a central role in the system: it monitors PV status and sends real-time information to the *Monitoring Center* system. The *Monitoring Plant Network* is based on the concept of “smart” PV modules, in which sensors and electronic components are set to control each panel or group of few panels; these *Smart Modules* are able to sense voltage, current and temperature and to send information through a wireless network at a service center (*Monitoring Center*).

The measured data will contribute to achieving both a local and a global vision of the behavior of the PV plant. These data can be integrated in the *Monitoring Center* structure with other relevant data, such as historical, season and meteorological data in order to analyze the efficiency of the PV plant. At the same time, these data will also allow users to develop new capabilities related to dynamic power point tracking at the *Smart Module* (or group of modules) level, automatic safety detection (fault prevention), night time theft monitoring and real time response. The overall architecture presents a modular and flexible structure based on open source technologies. As a result, it will be easily adapted to any context and environment.

This paper will mainly focus on the description of the sensors attached to every solar cell (and their associated circuitry) and on the protocols established to make that information reach the *Monitoring Center*, where it will be appropriately dealt with.

## 3. System Description

The correct performance of the whole system relies on the operation of the modules attached to each solar cell in order to collect the relevant information and send it to the *Monitoring Center*. These modules are supplied from the PV panel itself and, therefore, must guarantee low consumption levels. A detailed description of the operation of these modules and an explanation of the communication protocol follow.

### 3.1. Smart PV Modules

Each panel or group of panels includes a *Smart Module* that measures some relevant magnitudes in the panels and sends this information to a *Central Node* placed in the tower supporting the arrays of panels. These modules will therefore include some sensors to measure the magnitudes of interest and some circuitry in charge of the transmission. The power supply for these electronic circuits will be obtained from the panels themselves, while guaranteeing that the modules keep working even under low-radiation conditions—or during the night. This involves the use of some sort of energy storing device that manages to keep the circuit working when the panels are not providing enough energy; supercapacitors [25–27] have been identified as an optimum component to achieve this goal. Moreover, these devices can be found in the literature associated to both communication systems [28] and photovoltaic panels [29]. The most relevant features of these devices as compared to batteries, which are the traditional storage systems, are their capability to supply high current peaks and their almost non-existent maintenance within 500,000 charge/discharge cycles (typical batteries endure less than 500 charge/discharge cycles).

#### 3.1.1. Power Supply from the PV Panel

**Figure 2.**
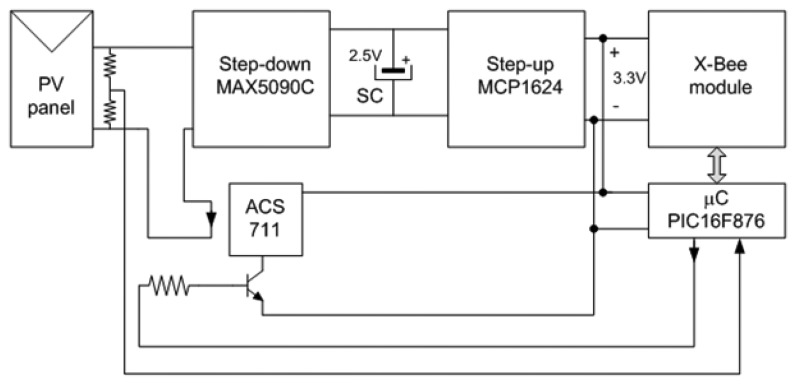
Schematic of the power supply and its connection to the Smart PV Module.

**Figure 3.**
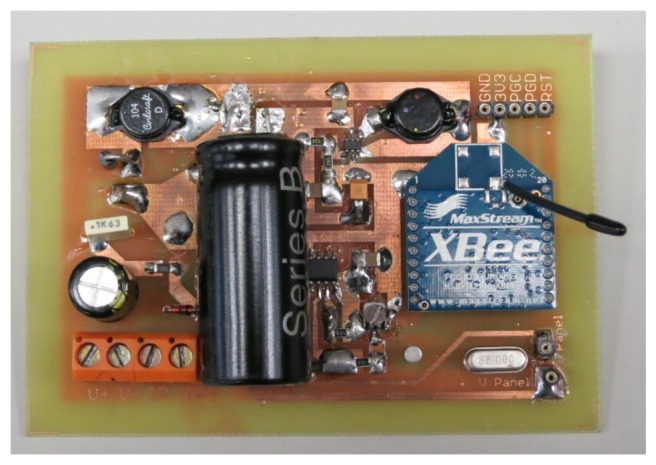
Supply system from PV panel to obtain the 3.3 V and the communication circuitry.

The power supply built includes two steps: one to charge the supercapacitor and another to supply the wireless communication stage as shown in Figure 2. A prototype of such a power supply and wireless communication system can be seen in Figure 3.

This power supply must guarantee that all the electronic circuitry is correctly supplied from the PV panel—even under low or no irradiation conditions—which means that it must be able to correctly charge the supercapacitor. Since the voltage provided by the PV panels is typically larger than the operating voltage of supercapacitors, a step-down converter must be included in the power supply. This converter controls the charge of the supercapacitor so that it is achieved in a reasonably short time.

Inversely, the operating voltage of the supercapacitor chosen (2.5 V) is lower than that required by the communication circuitry to work properly, namely 3.3 V. Therefore, a step-up converter is also necessary after the supercapacitor [30]. It is very important that this converter optimize the use of energy so as to make sure the supercapacitor stores enough energy to make the communication system operate during the night.

Although it is obvious that, as far as energy production is concerned, no useful information will be delivered during the night, the system is still operating when the sun is gone for safety purposes—protection against possible theft of the panels. In the same way, all the circuitry will be switched off during the idle periods to reduce consumption.

#### 3.1.2. Communication System

**Figure 4.**
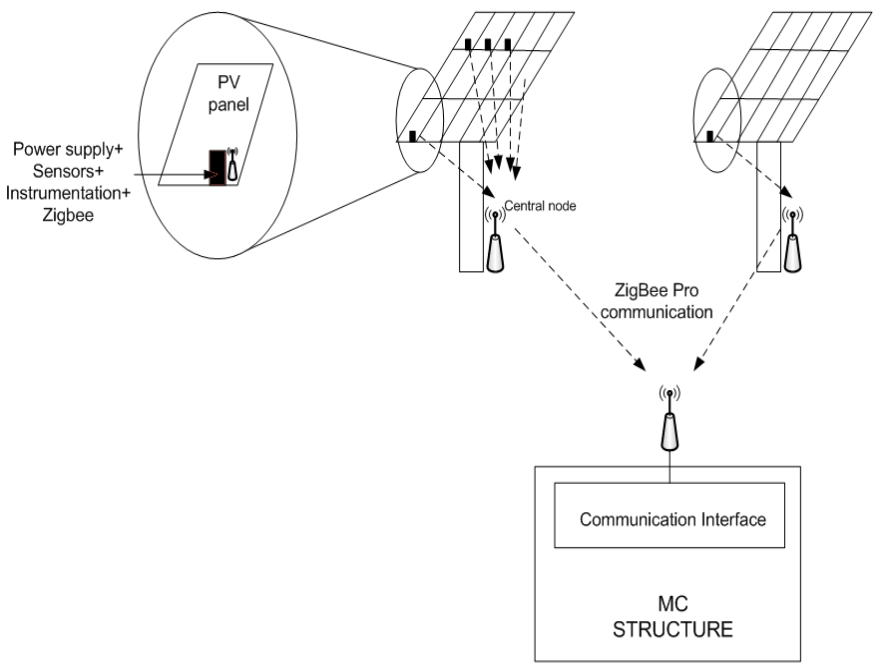
General structure of the wireless monitoring system.

Two different wireless communications can be identified in the solar plant (see Figure 4):

(a). Communication within the *Monitoring Plant Network: Smart Modules* send information to a *Central Node* on their tower; and
(b). Communication between these *Central Nodes* and the communication interface in the *Monitoring Center*.

In order to establish the communication between *Smart Modules* and the Central Node, each PV panel includes an XBee 802.15.4 low-power module from Digi [31] and an 8-bit PIC microcontroller from Microchip [32]. This node will also include all the circuitry in charge of sensing the magnitudes of interest. In the prototype, a current sensor from Allegro (ACS711 [33]) suitable to measure currents up to 12.5 A is included.

The Central Node (Figure 4 again) also consists of an XBee 802.15.4 low-power module and a PIC microcontroller, but it includes as well an XBee-PRO 802.15.4 extended-range module so that the communication with the *Monitoring Center* can be implemented.

Finally, an additional XBee-PRO 802.15.4 extended-range module and a PIC microcontroller will be required for the communication interface in the *Monitoring Center*. A device making it possible to connect this interface to a PC (such as a MAX232 [34] or FTDI FT245 [35], for instance) is also necessary.

### 3.2. Communication Protocol

In order to gather information from all the panels in the solar farm, a tree-structure using XBee modules is implemented. XBee modules have been chosen because they are easy to work with and have proven adequate performance in different kinds of applications [36–38].

There is a *Monitoring Center* attached to a central computer that stores and displays relevant data from all the panels. The *Monitoring Center* is aware of the solar farm distribution: how many towers there are, how many panels in each tower, *etc.* This information must be passed on to the other nodes during the setup stage. Therefore, it is the *Monitoring Center* that must be powered first, *i.e.*, before connecting the Central Nodes in the towers or the so-called *Smart Modules*.

**Figure 5.**
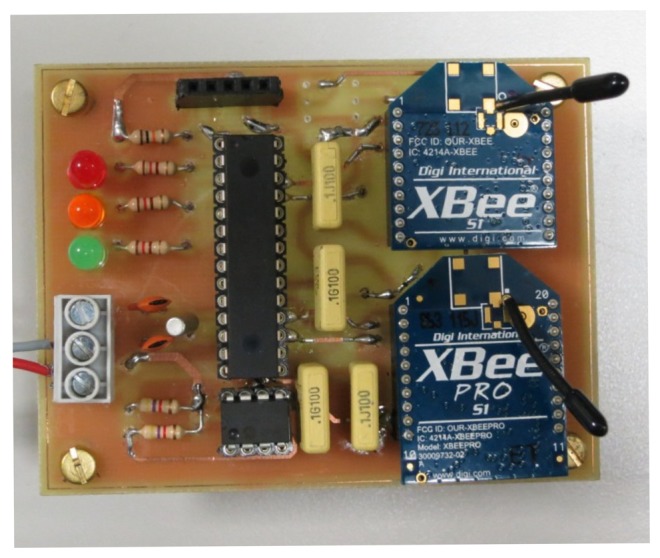
Central node prototype.

Once the *Monitoring Center* is powered, the towers' Central Nodes must be connected one at a time. This will allow the coordinator to identify them and assign them an individual name as well as the most adequate communication channel (CH) and network identifier (PAN). Other relevant information such as number of panels in the tower is also passed over from the *Monitoring Center* to the Central Nodes (Figure 5). In this way, all the towers are connected to the coordinator and ready to provide it with the information requested.

The following step is similar to the one just described; only it takes place in each of the towers in the solar farm. Each Central Node “knows” how many PV panels are associated to its tower, and a process begins by means of which all the *Smart Modules* are named as they are connected (one at a time). An appropriate communication channel and a PAN are also defined so that information can be exchanged as reliably as possible. A protocol that prevents nearby towers from using the same communication channel is also implemented to avoid interference. However, even if this was not done, having all the nodes named differently would guarantee that no cross-talking is produced.

After the configuration process is complete, the Central Nodes will periodically request each of its *Smart Modules* to send data (polling). This information will be stored until requested by the *Monitoring Center*. Finally, all the data are transferred to the main computer, where they are stored, displayed and/or analyzed. All the processes outlined above are associated to protocols and digital streams that will be briefly commented on later.

## 4. Operation of the System Implemented

**Figure 6.**
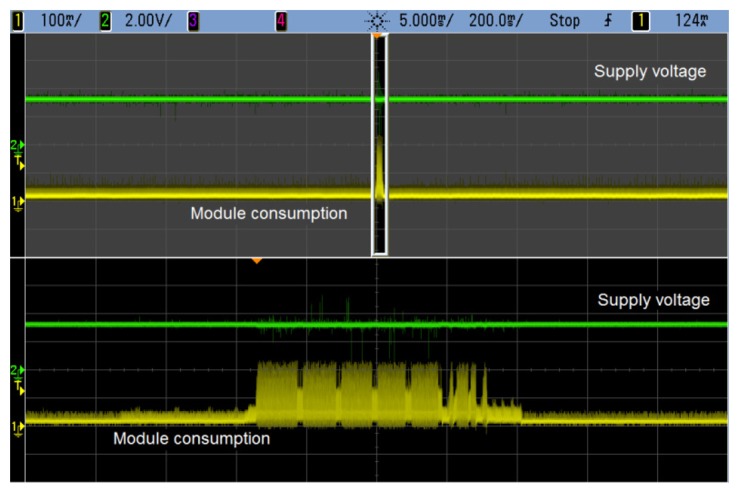
Zoom-in of the transmission interval. The output of the power supply remains stable all throughout this period.

Supply of the *Smart Modules* is a key issue towards the adequate performance of the system. As well as output voltage stability, one of the most important features of the power supply implemented is energy storage. Since the system must keep sending information during the night, the supercapacitor is selected to provide the energy required. Figure 6 shows typical current of the *Smart Module* during transmission of data, which represents an energy consumption that can be estimated to be some 185 mJ (the module is supplied at 3.3 V and an average current of 80 mA during 700 ms has been considered).

During the night, the system is sending information only for safety sake. Thus, it has been established that these packages of data be sent only every 4 min. Taking this into account, the supercapacitor chosen must be capable of supplying the energy required to guarantee operation during the night: 185 mJ every 240 s during 24 h, which results in 67 J (note a 24-hour period has been considered although only night operation is to be guaranteed). Once the energy to be provided by the supercapacitor has been estimated, the value of this component can be obtained by applying the expression of the energy stored in a capacitor:

(1) $$W=\frac{1}{2}\cdot C\cdot V^{2}$$

In this case, since the supercapacitor has a voltage of 2.5 V across its terminals, the value required to store 67 J is 21.4 F. A 25-F supercapacitor was selected for the preliminary prototype.

This power supply can also be used with any other sensors attached to PV panels. Only the value of the supercapacitor might need to be changed depending on the total energy demanded by the sensors included in the design.

The other key part in the system is the communication protocol. Two stages can be identified in this protocol: the system identification during start-up (or, as referred to in [18], synchronization of the WSN) and the steady-state performance during which the information is transferred through the network. This is described below.

### 4.1. Node Identification

In a WSN likely to include many *Smart Modules*, synchronization is a key issue. Appropriate identification of each of the nodes must be carried out so that problems do not arise later during steady-state operation. Thus, a protocol is defined that detects all the nodes in the system, provides them with a unique name and clearly identifies their location in the PV plant.

All the communication modules have predetermined default values when they are first powered up. This makes it possible to establish the initial communication between all of them. An example of a possible identification of two Central Nodes by the *Monitoring Center* is sketched in Table 1, which shows how two Central Nodes are detected on the default network (PAN = 1,000/CH = 10) and provided with new parameters to operate on the network to be used (PAN = 335/CH = 12).

A similar procedure will be followed to have each of the Central Nodes identify all the *Smart Modules* in their tower. From this point on, polling will be implemented so that the information captured at the *Smart Modules* in the PV panels can be sent over to the main computer in the solar farm.

### 4.2. Polling

Table 2 sketches the procedure followed to have a Central Node obtain the information gathered by the two *Smart Modules* in its tower.

*Smart Modules* sleep between two transmissions in order to reduce power consumption. Information provided during start-up (such as number of panels in the tower or information to send in each transmission) allows them to determine how long they can sleep so that they can wake up in time to meet the request from their Central Node.

The stream containing the information sent by the panels to the Central Node includes a header, number of data bytes in the stream, number of panel sending the information, values measured (in a pre-established order) and checksum:

HeaderN. bytesPanel IDVal.1Val.2Chksum


A similar procedure is followed by each of the Central Nodes to send the information to the *Monitoring Center*. In this case (two PV panels associated to the Central Node) the stream could look like this:

HdrNo. bytesTower IDPanel IDVal 1Val 2Panel IDVal 1Val 2Chk sum


### 4.3. Storing Information in the Computer

The *Monitoring Center* will carry out a polling similar to the one described above. After fulfilling the monitoring, storing and whatever other tasks that might be established for the system, a request from the computer will be sent to the *Monitoring Center* so that all the information available is transferred to the PC.

Up to five possible streams have been defined for the communication between the *Monitoring Center* and the main computer. All of them have a structure consisting of a header plus a data section (when necessary). The information will be transmitted using ASCII characters. The five stream types are:

- NACK. Indicates that the last stream was not correctly received.
- ACK. Indicates that the last stream was correctly received.
- DAT. This is a data stream.
- NDAT. Stream used by the overall coordinator to let the computer know there are no more available data.
- ENABLED. Used by the computer to request data from the overall coordinator.

## 5. Results and Discussion

The results included in this section are mainly focused on the correct performance of the power supply included in the *Smart Modules*. Some comments related to the communication protocol will also be provided.

### 5.1. Power Supply Performance

**Figure 7.**
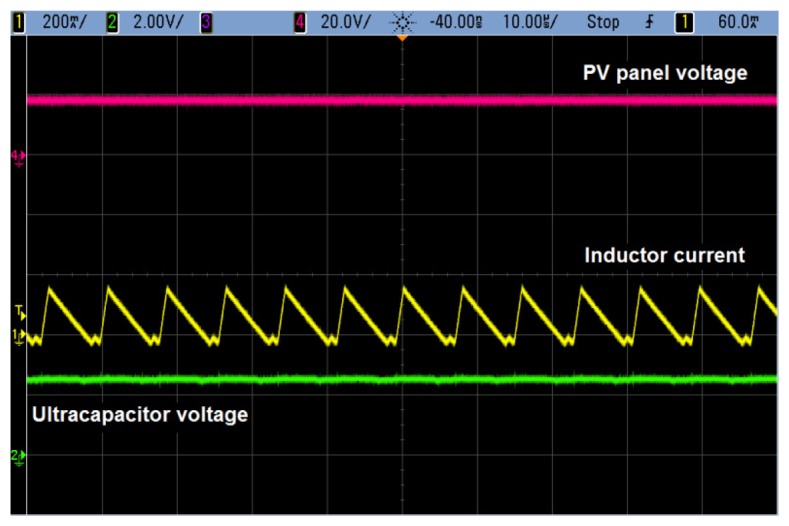
Waveforms associated to the operation of the step-down converter.

As already indicated, a step-down converter is needed in the power supply to charge the supercapacitor (up to 2.5 V) from the voltage delivered by the PV panel. This goal is achieved by including an integrated circuit by Maxim (MAX5090C [39]), which gives rise to the waveforms shown in Figure 7 during the charging process. In this figure, the capacitor has already reached its maximum voltage.

**Figure 8.**
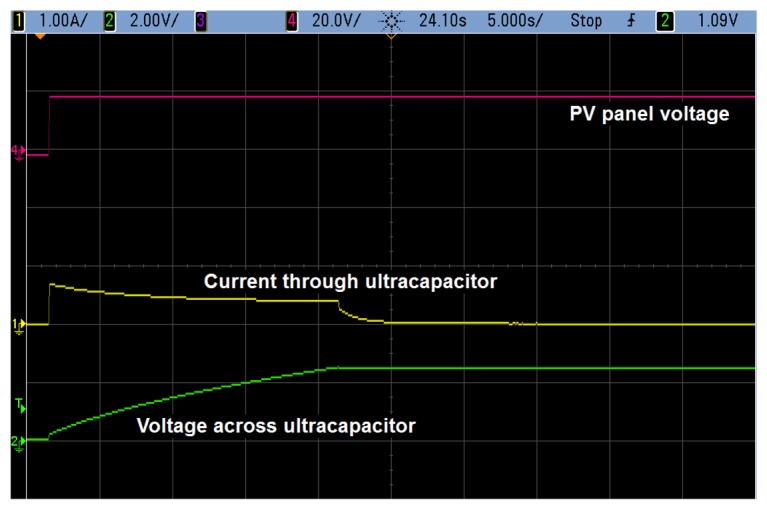
Initial charging of the supercapacitor. Considering a PV panel voltage of 20 V, it takes less than 20 s to have the supercapacitor fully charged.

Charging of the supercapacitor is achieved within a reasonable time interval. Figure 8 shows the waveforms corresponding to the initial charging process assuming that the supercapacitor is initially discharged and reaches 2.5 V. The time required for this is some 20 s assuming a maximum panel voltage of 20 V. Notice that this process will only take place the first time the system is connected to the PV panel. From then on, the system succeeds in keeping the supercapacitor almost fully charged—as long as there is enough sunlight.

Once the voltage across the supercapacitor has reached a minimum value, the step-up converter starts operating and producing a constant supply voltage. In the prototype developed, this voltage has been chosen to be 3.3 V, since that is a requirement of the XBee module and can also be used with the microcontroller. In this case, Microchip's MCP1624 integrated circuit [40] has been selected to generate the 3.3-volt voltage. In order to determine whether the performance of the power supply was adequate, it was connected to a preliminary prototype of a communication module.

**Figure 9.**
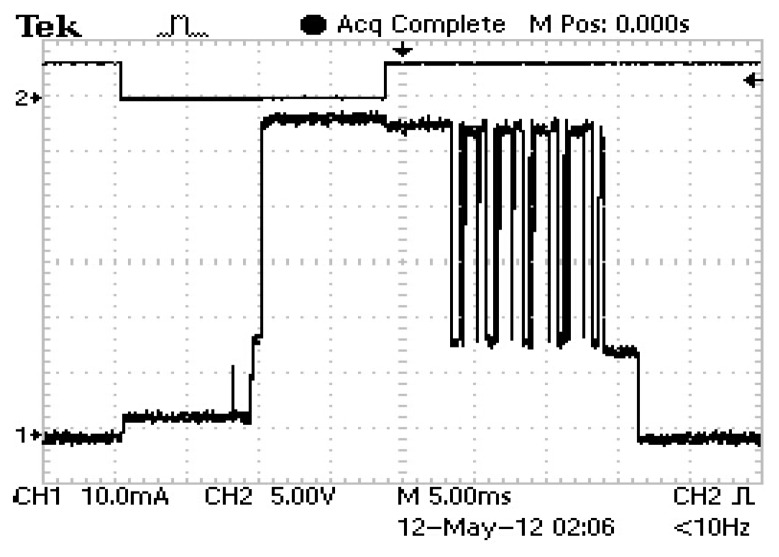
Current consumption during transmission (Channel 1). The signal on top is the wake-up period for the microcontroller in the *Smart Module*.

The consumption of the *Smart Module* is not constant. Most of the time it will be idle until the time comes to send the information to the Central Node. At this instant, the device wakes up from sleep and its consumption increases. This could be seen in Figure 6, which showed that the power supply implemented is stable during these transitions from sleep to active mode and vice versa. Figure 9 shows a close up of the current consumption where the wake-up signal can be seen.

**Figure 10.**
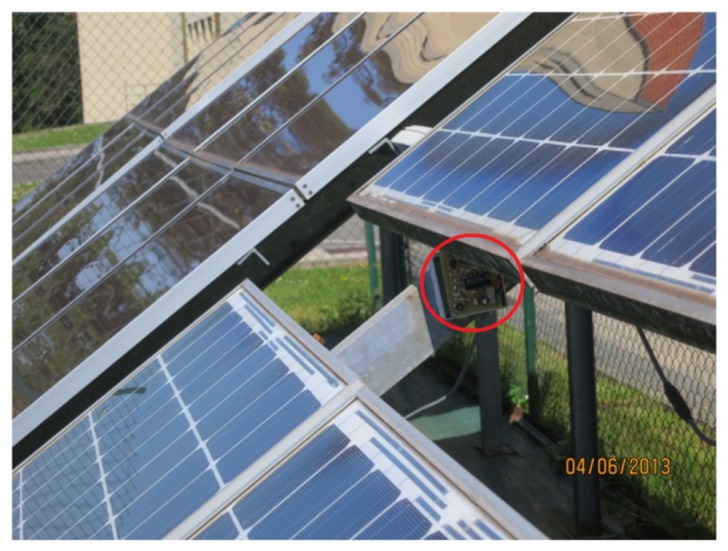
*Smart Module* attached to a PV panel during the tests.

**Figure 11.**
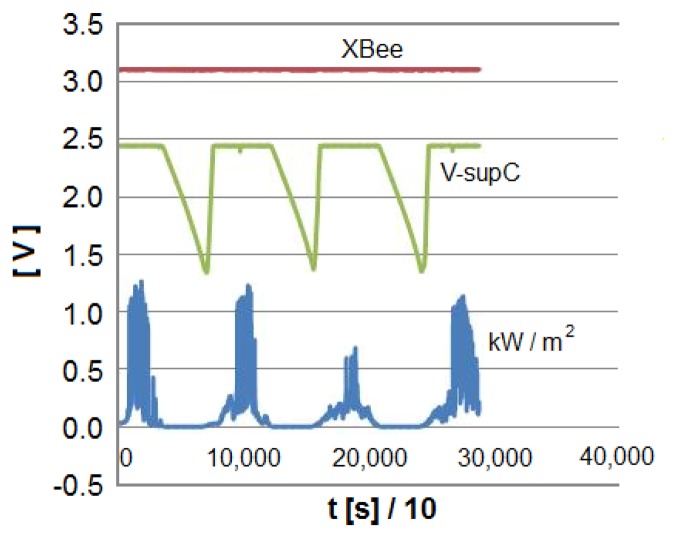
Evolution of solar radiation, supercapacitor voltage and XBee supply voltage during four days.

The supply for the XBee system has been tested during some months without power cuts in a small-scale PV plant available at our facilities. Figure 10 shows one of the *Smart Modules* designed attached to a photovoltaic panel whose current and voltage were being monitored. As indicated above, this *Smart Module* was supplied by the circuitry designed. Figure 11 illustrates the appropriate operation of the power supply by showing a period of four working days. All throughout this time, the voltage required by the system (labeled XBee in the Figure) is kept constant. Note also that the voltage across the supercapacitor never goes under 1.3 V, which means that 27% of the initial energy is still stored in the ultracapacitor. The low solar radiation present during the test should also be noticed.

### 5.2. Communication Protocol

The protocol implemented in the three blocks of the system (*Smart Modules*, Central Nodes and *Monitoring Center*) has been checked in the lab by means of preliminary prototypes that represented a network consisting of one *Monitoring Center* and two Central Nodes: one including two *Smart Modules* and the other one with only one *Smart Module*. This system succeeded in identifying all the nodes during start-up, after which the *Smart Modules* periodically send the information to the *Monitoring Center*. Extension to a larger system only requires that the system parameters be changed.

**Figure 12.**
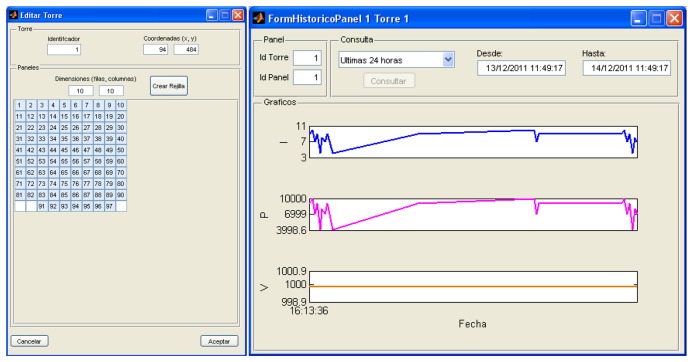
Matlab monitoring environment.

Also, a Matlab-based environment has been developed for testing purposes that complies with the features described above. This environment allows users to define the size of the PV plant considered and plot the evolution of relevant magnitudes associated to the whole plant, to a given tower or even to a simple panel. Figure 12 shows an example of the windows created for this purpose, where 97 PV panels from a solar tower are monitored. The window on the right allows users to define a particular period of time during which the power, voltage and current evolutions of any selected PV panel can be plotted.

## 6. Conclusions

This paper fully describes a WSN suitable for inclusion in photovoltaic plants in order to improve efficiency and optimize energy production. This is achieved by developing smart sensor nodes attached to each PV panel in the plant and defining a communication protocol that succeeds in taking all the information related to the panels to a *Monitoring Center*, where it is displayed and conveniently analyzed. A start-up procedure to identify all the nodes in the WSN has also been presented.

The sensors attached to each panel are supplied power from the panels themselves. Thus, a power supply that obtains energy from the panel and stores it during the night so that information can still be gathered and sent to the *Monitoring Center* (for safety purposes, mainly) has been designed. Supercapacitors have been used as energy storage devices instead of the traditional batteries in order to take advantage of their high cycling capability and their fast charge process. Such a power supply can be used by any other sensors that get their energy from PV panels and must still operate during the night.

Preliminary prototypes demonstrating the feasibility of the system described have been implemented and tested. A MATLAB application has been developed to define the preliminary configuration of the communication network used to collect the data from each PV panel.

Future work is related to cost-optimization of the *Smart Modules* so that the system becomes more attractive to developers of PV plants. Also, and even though authors preferred defining a self-contained system that did not depend on third-party products, a system based on storing information in the cloud might be considered.

## Figures and Tables

**Table 1.** Node identification at start-up.

**Monitoring Center**	**Central Node 1**	**Central Node 2**
Turn-on, Addr = 0, Pan = 1,000/Ch = 10		
Gets data from PC, num_towers←2		
	Turn-on, Addr = 1, Pan = 1,000/Ch = 10	
Detects new module, found←1		
Starts communication on default PAN, Addr0⇒Addr1		
Sends new parameters	Gets new parameters, Addr←001, Pan←335/Ch←12	
Waits for new modules, found < num_towers		
		Turn-on, Addr = 1, Pan = 1,000/Ch = 10
Detects new module, found←2		
Starts communication on default PAN, Addr0⇒Addr1		
Sends new parameters		Gets new parameters, Addr←002, Pan←335/Ch←12
Identification complete, Found = num_towers		
Moves to active PAN, Addr←000, Pan←335/Ch←12		

**Table 2.** Polling during normal operation.

**Central Node**	**Smart Module 1**	**Smart Module 2**
		
…	…	…
		
	Wakes up	*Sleeping Time*
	
Sends request *panel*←*1*	Receives request	
	
Receives information *IN*←*Stream*	Sends information *OUT*←*Stream*	
	
Sends ACK	Starts Timer and sleeps	
	
Stores information	*Sleeping Time*	
	
		Wakes up
	
Sends request *panel*←*2*		Receives request
	
Receives information *IN*←*Stream*		Sends information *OUT*←*Stream*
	
Sends ACK		Starts Timer and sleeps
	
Stores information		*Sleeping Time*

*[Sends information to coordinator]*		
	
	Wakes up	
	
Sends request *panel*←*1*	Receives request	
	
…	…	…
